# Infectious corneal ulceration: a proposal for neglected tropical disease status

**DOI:** 10.2471/BLT.19.232660

**Published:** 2019-11-01

**Authors:** Lawson Ung, Nisha R Acharya, Tushar Agarwal, Eduardo C Alfonso, Bhupesh Bagga, Paulo JM Bispo, Matthew J Burton, John KG Dart, Thuy Doan, Suzanne MJ Fleiszig, Prashant Garg, Michael S Gilmore, David C Gritz, Linda D Hazlett, Alfonso Iovieno, Vishal Jhanji, John H Kempen, Cecilia S Lee, Thomas M Lietman, Todd P Margolis, Stephen D McLeod, Jod S Mehta, Darlene Miller, Eric Pearlman, Lalitha Prajna, N Venkatesh Prajna, Gerami D Seitzman, Swapna S Shanbhag, Namrata Sharma, Savitri Sharma, Muthiah Srinivasan, Fiona Stapleton, Donald TH Tan, Radhika Tandon, Hugh R Taylor, Elmer Y Tu, Sonal S Tuli, Rasik B Vajpayee, Russell N Van Gelder, Stephanie L Watson, Michael E Zegans, James Chodosh

**Affiliations:** a Department of Ophthalmology, Massachusetts Eye and Ear, Harvard Medical School, 243 Charles Street, Boston, Massachusetts 02114 United States of America (USA).; b Francis I. Proctor Foundation, University of California San Francisco, San Francisco, USA.; c Dr Rajendra Prasad Centre for Ophthalmic Sciences, All India Institute of Medical Sciences, New Delhi, India.; d Bascom Palmer Eye Institute, University of Miami Miller School of Medicine, Miami, USA.; e Tej Kohli Cornea Institute, L.V. Prasad Eye Institute, Hyderabad, India.; f International Centre for Eye Health, London School of Hygiene & Tropical Medicine, London, England.; g Moorfields Eye Hospital, London, England.; h School of Optometry, University of California, Berkeley, USA.; i Wilmer Eye Institute, Johns Hopkins University School of Medicine, Baltimore, USA.; j Department of Ophthalmology, Visual and Anatomical Sciences, Wayne State University School of Medicine, Detroit, USA.; k Department of Ophthalmology and Visual Sciences, University of British Columbia, Vancouver, Canada.; l Department of Ophthalmology, University of Pittsburgh School of Medicine, Pittsburgh, USA.; m MCM Eye Unit, MyungSung Medical School, Addis Ababa, Ethiopia.; n Department of Ophthalmology, University of Washington School of Medicine, Seattle, USA.; o Department of Ophthalmology and Visual Sciences, Washington University in Saint Louis School of Medicine, Saint Louis, USA.; p Singapore National Eye Centre, Duke-NUS Graduate Medical School, Singapore.; q Institute of Immunology, University of California, Irvine, USA.; r Aravind Eye Hospital, Madurai, India.; s Jhaveri Microbiology Centre, L.V. Prasad Eye Institute, Hyderabad, India.; t School of Optometry and Vision Science, University of New South Wales, Sydney, Australia.; u Melbourne School of Population and Global Health, University of Melbourne, Melbourne, Australia.; v Department of Ophthalmology and Visual Sciences, University of Illinois at Chicago, Chicago, USA.; w Department of Ophthalmology, University of Florida, Gainesville, USA.; x Royal Victorian Eye and Ear Hospital, University of Melbourne, Melbourne, Australia.; y Save Sight Institute, University of Sydney Medical School, Sydney, Australia.; z Department of Surgery (Ophthalmology), Geisel School of Medicine at Dartmouth, Hanover, USA.

In ophthalmology, the designation of trachoma, onchocerciasis and leprosy as neglected tropical diseases (NTDs) has sustained efforts to combat these blinding conditions worldwide. Over the past 50 years, NTD designations have enabled the joining of political, social and economic forces to promote research and interventions for diseases that overwhelmingly affect the 3 billion people who subsist on less than 2 United States dollars (US$) a day.^1^ The global public health landscape is still dominated by focus on human immunodeficiency virus (HIV), tuberculosis and malaria. However, NTDs are now increasingly recognized as important causes of morbidity and mortality in low-income settings, perpetuating stigma and social isolation, with many NTDs leading to disfiguring complications. In international public health diplomacy, formal disease recognition is essential. The pursuit of this recognition drives proposals from World Health Organization’s (WHO’s) Member States to include additional diseases in the list of NTDs. The intention is to strengthen the development of partnerships, epidemiological frameworks and commitment of resources to achieve the aims set by the sustainable development goals.^2^

Despite ongoing efforts to end preventable blindness, infectious corneal ulceration still receives insufficient attention for reasons that are unclear. The condition occurs when microbes from the environment invade the cornea to produce inflammation which in turn leads to ulceration. A study conducted two decades ago estimated that over 1.5 million people worldwide will develop blindness from infectious corneal ulceration each year,^3^ a number that most likely underrepresents the true scale of this disease. Our combined clinical experience suggests that an even greater number will experience visual disability, mostly unilateral, that will fall just short of current WHO-defined measures of blindness. Nonetheless, infectious corneal ulcers are the most common cause of non-trachomatous corneal opacification, and the fifth leading cause of blindness overall, responsible for up to 3.5% (36 million) of all blind persons as of 2015.^4^ Most of this burden falls on low-income countries, where the etiology, epidemiology and patterns of clinical presentation are distinct from those in high-income countries, and where it is not uncommon for loss of vision in one eye to portend future loss in the other. 

Children who are affected by infectious corneal ulceration face a lifetime of increased general morbidity, with the onset of visual impairment also strongly associated with increased risk of childhood mortality.^5^ With the burden of corneal ulceration in low-income countries now surpassing the traditional blinding diseases in magnitude,^6^ the inclusion of infectious corneal ulcers among currently recognized NTDs could be the first step in addressing the needs of those affected. Should this inclusion not happen, re-examining infectious corneal ulceration through the NTD paradigm may nonetheless inspire the necessary collective discussion and action as we move beyond the global initiative VISION 2020.^5^

Viewing infectious corneal ulcers as an NTD can help us analyse how we can apply elements of historically successful public health campaigns in ophthalmology to this disease. The successes of the SAFE (surgery, antibiotics, cleanliness and environmental change) strategy for trachoma, as well as the administration of yearly or biannual ivermectin for onchocerciasis, remain driven primarily by the robust partnerships created between local community health centres in underserved areas, government agencies, nongovernmental organizations and pharmaceutical companies. Between 1990 and 2013, these programmes contributed to dramatic declines in the global prevalence of trachoma and onchocerciasis.^7^

Infectious corneal ulceration disproportionately affects farming-based societies across the WHO Regions of Africa, the Americas, South-East Asia and Western Pacific. Agricultural workers are at increased risk for minor ocular trauma, which in turn can lead to infection with pathogens ubiquitous in soil, plant matter and water. Without prompt medical attention, even a minor corneal abrasion can develop into a blinding corneal ulcer. The severity of infectious corneal ulceration is also worsened by vitamin A deficiency, commonly the result of poor nutritional status, and independently associated with corneal ulceration and blindness. Additionally, the emergence of antimicrobial resistance and the harmful use of widely available traditional eye medicines^8^ and topical corticosteroids all may lead to poorer disease outcomes. Considering the social, economic, environmental, and cultural risk factors for infectious corneal ulceration and the number of individuals living in high-risk areas, we should not expect the incidence of this disease to decrease without population-based interventions.

One solution to the problem of infectious corneal ulceration may lie in the delivery of a simple, safe and effective community-based strategy. NTDs are considered preventable and treatable. The main etiologies of infectious corneal ulcers, including bacteria, fungi and parasites, are often clinically indistinguishable and any attempt to reduce their burden must take the broad range of causative organisms into consideration. 

Past attempts to reduce the burden of infectious corneal ulcers have involved community health promotion campaigns and the mobilization of trained community eye workers to reach remote or rural areas. In 2018, study subject recruitment was completed for the Village-Integrated Eye Worker trial, a cluster-randomized study in Nepal that aims to evaluate the effectiveness of 1% chloramphenicol and 1% itraconazole ointment to prevent corneal abrasions from becoming corneal ulcers.^9^ Conducted on the precedent of similar, but non-randomized studies in India and Nepal,^10^^,^^11^ the trial may perhaps provide the necessary efficacy, cost and feasibility data, and therefore the scientific and clinical grounds, for what is a simple intervention. 

An important distinction between the aforementioned trial and other public health campaigns, such as the SAFE (surgery, antibiotics, facial cleanliness and environmental change) strategy is that the former is an example of primary prevention and seeks to counteract the onset of the condition. Clearly, the possibility of establishing cost–effective, community-driven models of eyecare exists, wherein pharmacological prophylaxis is augmented by educational initiatives and distribution of protective eyewear. The Village-Integrated Eye Worker trial may provide an insight into how ophthalmologists, eye health-care workers and public health practitioners collaborate to prevent infectious corneal ulceration in low-income countries.

Beyond the prospect of mobilizing the necessary resources that may come with NTD recognition, rethinking infectious corneal ulceration in this manner may improve our current poor understanding of its global epidemiology. For infectious corneal ulcers, even the most basic data, incidence, prevalence, contribution to disability-adjusted life years, and loss of productivity indices, all remain unknown. The only data available comes from outdated population surveys, which suggest that South and South-East Asia are the epicentres of disease, with reported incidences of 113 per 100 000 persons in India,^12^ 339 per 100 000 in Bhutan,^8^ 710 per 100 000 in Burma^8^ and 799 per 100 000 in Nepal.^11^

Mapping of key endemic areas and establishment of disease surveillance systems are needed, particularly in areas where no such data exists. Furthermore, the historical inclusion of infectious corneal ulcers as a major cause of corneal opacity has not provided the detail required for accurate epidemiological study. Separating infectious corneal ulcers from these blanket descriptions may provide the necessary information to generate interest among global and regional health agencies. 

Innovations in corneal ulcer care worldwide may include improvements in the treatment of frequently recalcitrant fungal ulcers that predominate in low-income countries, as well as the selection of antimicrobials according to regional pathogen distributions and the development of novel therapeutic agents to minimize corneal scarring of sufficient severity to impact vision. Strategies to reduce the burden of infectious corneal ulceration will also involve tailoring sustainable public health interventions according to the specific needs of communities where such care is needed most.

To eliminate avoidable blindness, we must address the burden of infectious corneal ulcers. With the results from trials such as Village-Integrated Eye Worker trial pending, we may soon have evidence of a practical and replicable demonstration of the value of corneal ulcer prophylaxis within resource-constrained settings. The relatively scarce attention given to infectious corneal ulceration does not reflect the impact of the condition on the most vulnerable, many of whom live in poverty. The classification of infectious corneal ulceration as an NTD would be timely and appropriate and would allow us to adequately address this relatively overlooked disease.
